# Pre-dispersal strategies by *Quercus schottkyana* to mitigate the effects of weevil infestation of acorns

**DOI:** 10.1038/srep37520

**Published:** 2016-11-22

**Authors:** Ke Xia, William L. Harrower, Roy Turkington, Hong-Yu Tan, Zhe-Kun Zhou

**Affiliations:** 1 Germplasm Bank of Wild Species, Kunming Institute of Botany, Chinese Academy of Sciences, Kunming, Yunnan, 650201, China; 2 Key Laboratory for Plant Diversity and Biogeography of East Asia, Kunming Institute of Botany, Chinese Academy of Sciences, Kunming, Yunnan, 650201, China; 3 Botany Department, and Biodiversity Research Centre, University of British Columbia, Vancouver, BC V6T 1Z4, Canada; 4 Quicken Loans Inc., Detroit, Michigan, 48226, USA; 5 Xishuangbanna Tropical Botanical Garden, Chinese Academy of Sciences. Menglun, Mengla, Yunnan, 666303, China

## Abstract

We investigated how pre-dispersal strategies may mitigate the effects of weevil infestation of acorns in a population of *Quercus schottkyana,* a dominant oak in Asian evergreen broad-leaved forests, and assess if weevil infestation contributes to low seedling recruitment. We counted the number of acorns produced, daily from the end of August to mid-late November for 9 years from 2006–2014. We also recorded the rate of acorn infestation by weevils and acorn germination rates of weekly collections. Annual acorn production was variable, but particularly low in 2011 and 2013. There was no trade-off between acorn production and acorn dry mass. However, acorns produced later in the season were significantly heavier. For most years: (i) the rate of weevil infestation was negatively density dependent (a greater proportion of acorns died with increased acorn density), (ii) the percentage germination of acorns was positively density dependent (proportionately more acorns germinated with increased density), and (iii) as the season progressed, the percentage of infested acorns declined while germination rates increased. Finally, (iv) maximum acorn production, percentage infestation and percentage germination were asynchronous. Although pre-dispersal mortality is important it is unlikely to be the primary factor leading to low recruitment of oak seedlings.

Pre-dispersal seed predation may cause the destruction of a great proportion of the seeds produced by plants and is often the main limitation of the number of seeds to be dispersed^1^,^2^,^3^. Consequently, plants have evolved a variety of traits that minimize the negative effects of seed predation such as antipredator defenses^4^, larger seeds^5^, irregular inter-annual phenology^6^ and production of seeds^7^,^8^,^9^ and premature abortion of infested seeds^5^. In addition, the presence and abundance of natural enemies influences the extent of pre-dispersal seed predation^10^. The aim of this study was to explore pre-dispersal losses of acorns of *Quercus schottkyana* caused by weevil infestation and to investigate adaptations by the oak to mitigate these losses.

*Quercus schottkyana (Cyclobalanopsis glaucoides* in the Flora of China^11^) is the dominant species of oak in the Asian evergreen broad-leaved forests in Southwestern China^12^,^13^. These forests contain more than 20,000 species of higher plants (6% of the world’s total) along with many unique animal species^14^. As the dominant species in this ecosystem, *Q. schottkyana* provides the major structural component for the forests of Yunnan Province and as such provides habitat for many other plants and animals; however seedlings of *Q. schottkyana* and other oaks are not very abundant^15^,^16^. Recruitment of *Q. schottkyana* from acorns is problematic and currently populations of *Q. schottkyana* mainly depend on recruitment by resprouting^15^,^17^. An understanding of the factors that influence the long-term survival of *Q. schottkyana* forests, particularly acorn production and their germination, is critical to the maintenance of these forests and subsequently the biodiversity they support. It is necessary, therefore, that we attempt to identify some of the factors that ultimately influence oak seedling establishment by initially investigating acorn production, pre-dispersal predation (mostly weevil infestation) and acorn germination.

Annual acorn production by *Q. schottkyana* is variable and one of the simplest explanations is that plants respond to variable weather conditions by producing more seed in good years^7^,^8^. However, the primary objective of our study was to investigate how variation in acorn production ultimately influences pre-dispersal predation which in turn may influence acorn survival and germination. The major pre-dispersal predators are weevils (*Curculio*, Coleoptera: Curculionidae) that infest acorns on the trees before the acorns fall to the ground. Infestation by weevils can damage up to 49% of an annual acorn crop and limit the availability of quality acorns for germination and seedling establishment^15^. Weevils are one of the major causes of declining regeneration of *Quercus* spp. in northwestern India^18^ and have been reported to infest or destroy up to 70% of acorns of *Q. crispula* in Japan^9^, and >90% of acorns in eastern hardwood forests in the US^19^,^20^. Despite the dominance and importance of *Q. schottkyana* to the integrity of these southern Chinese forests and the region’s biodiversity, few studies have reported acorn production and rates of weevil infestation by any of the oak species in the subgenus *Cyclobalanopsis*^15^,^21^ and their data are only for 1 or 2 years.

We monitored total weekly acorn production patterns, percentage infestation of acorns by weevils and percentage germination of acorns in *Q. schottkyana* over a 9-year period from 2006–2014 to determine if weevil infestation has a significant impact on the number of acorns that survive and subsequently germinate. Our data identify three strategies used by *Q. schottkyana* to mitigate the effects of pre-dispersal weevil infestation.

## Results

### Acorn production

**Figure 1:**
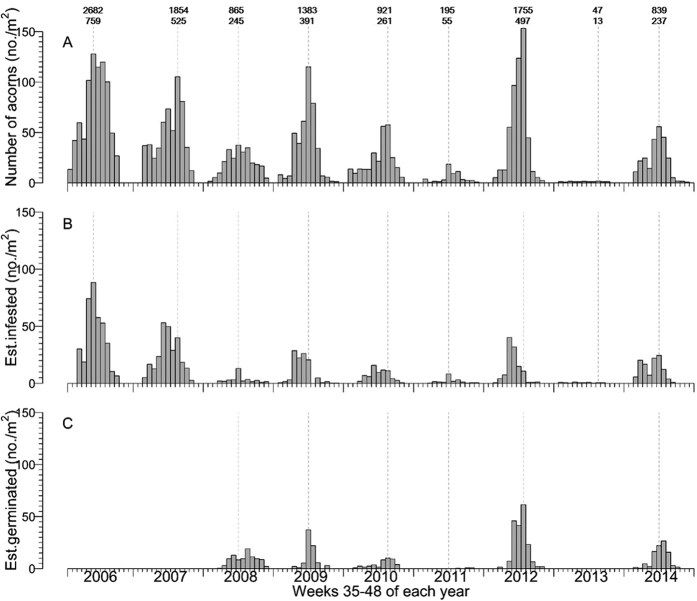
Weekly acorn production and weekly estimated infestation and germination numbers by *Quercus schottkyana* from 2006–2014. The timing of peak acorn production is indicated with a vertical dashed grey line. Germination tests were not conducted on acorns collected in 2006, 2007 and 2013 (in 2013 too few acorns were produced to permit germination tests). The two numbers above the bars in Fig. 1A are: (upper) total acorn production - the total number of acorns collected in 50 circular basins each 30 cm diameter and a total area of 3.54 m^2^; (lower) acorn density - calculated as (total number of acorns produced/3.54 m^2^).

Annual acorn production was variable but was particularly low in 2011 and 2013. This resulted in significant between-year variation in acorn production (χ^2^_2,10_ = 179.7, p < 0.001; Fig. 1A; Supplementary Table S1.1). Annual production ranged from a low of 13 acorns.m^−2^ in 2013, to a high of 759 m^−2^ in 2006 (Fig. 1A). Acorn production occurred between late August and mid-late November (weeks 35–48 in a 52 week year) and the timing of peak acorn production varied only slightly between years typically peaking in late October (week 40 in 2006, week 41 in 2008, 2009, 2011, 2014, week 42 in 2012 and week 43 in 2007, 2010 and 2013). When data for all nine years are combined there is weekly variation in acorn production (χ^2^_2,4_ = 182.52, p < 0.001, Supplementary Table S1.2) with a production peak during week 41.

**Figure 2:**
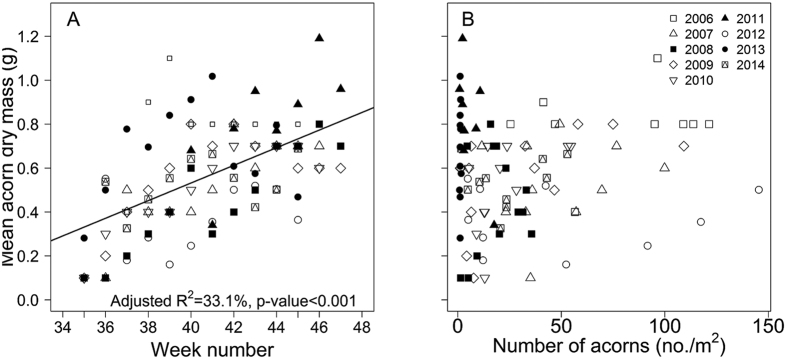
Relationship between dry mass (g) of acorns of *Quercus schottkyana* from 2006–2014, (**A**) throughout the acorn production season and (**B**) and the weekly density of acorn production.

The quality of acorns differed both within and between each of the nine years. Most acorns are mature when dropped from the tree, but acorns dropped early in the season (weeks 35–37, late August to early September) had a dry mass <0.4 g, and up to 59% were typically immature and were aborted. These immature acorns had small or sometimes no cotyledons inside the pericarp upon collection. There was a difference in the size of acorns between years (χ^2^_3,11_ = 51.38, p < 0.001, Supplementary Table S1.3). When we accounted for variation in acorn size between weeks, acorns produced later in the season were significantly heavier (Fig. 2A; χ^2^_3,4_ = 46.52, p < 0.001; Supplementary Table S1.4, Fig. S1A,). However, by comparing models with and without the total number of acorns produced, we found no significant relationship between acorn size and the total number of acorns produced between weeks with the variation in acorn size (Fig. 2B, χ^2^_4,5_ = 1.413, p = 0.234, Supplementary Table S1.5, Fig. S1B,). Therefore, there was no trade-off between acorn production and acorn dry mass and heavy acorns were produced in both high and low production weeks and years.

### Infestation rates by weevils

**Figure 3:**
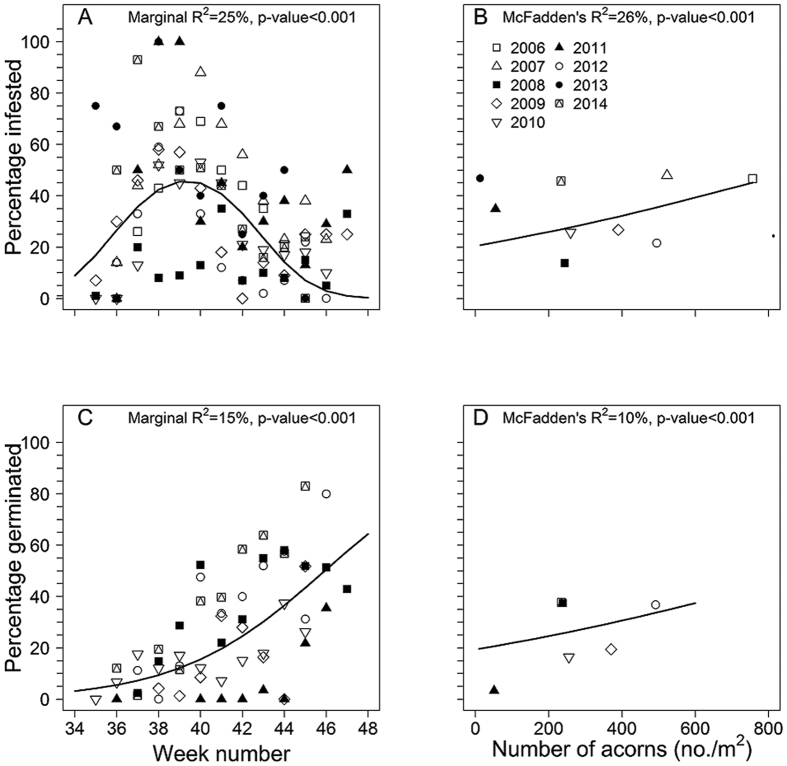
Relationship between the percentage infestation (**A,B**) and percentage germination (**C,D**) of acorns of *Quercus schottkyana* dependent upon (**A,C**) the time during the acorn production season, and (**B,D**) the density of yearly acorn production.

There was variation in infestation rates of acorns by weevils between years (Fig. 1B, χ^2^_3,11_ = 41.65, p < 0.001, Supplementary Table S2.1) and weeks. Although there was an increase in infestation rate between weeks 34 and 39, after week 39, infestation rates decreased over time (Fig. 3A, χ^2^_2,4_ = 626.07, p < 0.001; Supplementary Table S2.2). When we account for difference in production between years, there was a significant negative density dependent relationship with an increasing rate of infestation by weevils as acorn production increased (Fig. 3B, χ^2^_1,2_ = 215.81, p < 0.001; Supplementary Table S2.3). On average 34% of acorns (SE = 0.05) were infested by weevils.

### Acorn germination

As with production and infestation, there was variation in germination rates between years (χ^2^_3,8_ = 31.06, p < 0.001, Supplementary Table S3.1) and between weeks (Fig. 1C, Supplementary Table S3.1). Percent germination increased throughout the season (Fig. 3C, χ^2^_2,3_ = 315.67, p < 0.001 Supplementary Table S3.2). There was a positive density dependent relationship with percentage germination of acorns increasing with increasing acorn density (Fig. 3D, χ^2^_1,2_ = 37.81, p = 0.001; Supplementary Table S3.3).

### Timing of acorn production, weevil infestation and germination

**Figure 4:**
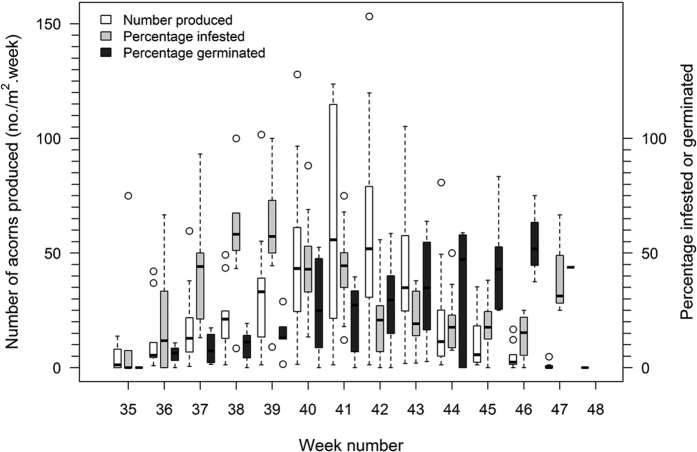
Acorn production of *Quercus schottkyana*, percent infestation, and percent germination by week of the year, combined for all years from 2006–2014.

When we combine data from 2006–2014 it is apparent that maximum acorn production (week 41), percentage infestation (week 39) and percentage germination (week 46) are not synchronous (Fig. 4) but in poor production years there was a tendency for infestation rates to remain high throughout the year (Fig. 1).

## Discussion

By conducting detailed weekly monitoring in a population of *Quercus schottkyana* for 9 seasons we have demonstrated links between acorn production, acorn dry mass, acorn quality, weevil infestation and germination which may have important implications for the persistence of this endemic oak. Our data demonstrate that *Q. schottkyana* has at least three pre-dispersal strategies that mitigate the effects of predation by weevils on its acorns. First, it produces large numbers of acorns; second, it produces poor quality acorns early in the season that satiates most of the weevils; third, it produces higher quality acorns later in the season that have a higher probability of germinating, i.e. a temporal refuge. Points two and three combined provide the possibility that populations of *Q. schottkyana* may be able to satiate both small and large seed predators which is an unusual trait given the widespread occurrence of a seed size - seed number trade-off 22,23 in individual plants. It is of interest that many characteristics of plant populations are the sum of those traits from the individuals comprising the population, for example, seed production, even though there is consistent and wide interindividual variation^24^,^25^,^26^,^27^,^28^. This in turn affects other seed traits such as seed size and various selectively important processes such as pre- and post-dispersal predation level. In higher production years, some acorns ‘escape’ infestation because of overproduction but these acorns are also safe from weevil predation late in the growing season when the more viable acorns are produced. Thus, the number of viable acorns that the population of trees produces is determined by the number of acorns produced by all trees, acorn quality and the timing of production. This method of predator satiation used here by *Quercus schottkyana* may be vulnerable in years when the population is unable to produce an abundance of acorns.

There are at least two major factors that influence acorn production and acorn quality, and ultimately determine the number of viable acorns available for germination – abiotic factors such as local variation in weather and biotic factors such as seed predators.

### Abiotic factors

One of the primary aims of our study was to document long term acorn production (9 years) at a fine scale (daily and weekly production); the study was not designed to determine causes of variability of acorn production but a discussion of some potential causes is useful. One of the simplest explanations for variable seed production is that plants respond to variable weather conditions by producing more seed in good years^7^,^8^. Weather conditions at the time of pollination can be good predictors of acorn crop size^29^. Acorn production is likely influenced by spring rainfall in southern China, but also by other factors we have not yet identified. Various weather characteristics, including temperature and rainfall, are widely considered as cues that influence acorn production^30^. Weather conditions that result in poor acorn production include extended periods of rain during the flowering period that reduce a tree’s ability to pollinate^29^ and tolerate drought^31^. This is corroborated here where the two lowest acorn crops (2011, 2013) occurred in dry years and 2011 was an extremely severe drought. Local weather influences pollination in some oaks through drought or temperature stress^31^ and also has a direct impact on the acorn crop size^32^. Pollination of *Q. schottkyana* generally occurs before mid-April^33^. After pollination, the warmer temperatures in May and June promote ovules and young fruit^34^. The warmer weather during spring time promotes megagametogenesis and embryogenesis and likely increases acorn production^34^.The local weather patterns especially during pollination can have a profound impact on yearly acorn production but this requires more research in our region.

### Biotic factors

Plants can increase the probability of seed viability and survival by episodically producing large crops of seeds thereby satiating seed predators, or by producing larger seeds. A number of studies investigating weevil attack on acorns have shown lower rates of infestation during years of high acorn production^35^. For example, 66% of *Q. liaotungensis* (a variety of *Q. mongolica*)^16^ and 86% of chestnut oak acorns^20^ were attacked by weevils during years of lower acorn production and only 29% and 26% in high production years. Indeed, a negative correlation between seed crop size and predation rate by seed insects has been reported for many temperate tree species^9^. This is contrary to our results where, during the nine years, the greater the density of acorns the higher the infestation rates. This negative density dependent relationship broke down in low-crop years. In years 2011 and 2013 when there was low acorn production, the percentage of infested acorns remained relatively high throughout the season and only 3% acorns (in 2011) were viable at the time of dispersal compared with that of 36% in 2012 with a 9 times higher acorn density. Yi and Yang^5^ also reported a negative density dependent rate of weevil attack on acorns of *Quercus aliena*.

Insect herbivory frequently leads to reduced seed size with potential effects on seed germination, seedling size and competitiveness^36^. Insect herbivores may also influence the evolution of seed size by inflicting a higher death rate on some sizes of seeds than on others. For example, the acorn weevil *Curculio glandium* kills a significantly greater proportion of small acorns than of larger acorns from the same tree^36^ a result entirely consistent with our data. Larger acorns in *Quercus ilex* had higher germination rates and seedling survival, accelerated germination timing and enhanced seedling growth^27^. Nevertheless, there was also a direct negative effect of acorn size on survival to predation, because large acorns were highly preferred by the main post-dispersal seed predators at the study site, wild boars and wood mice. Many studies show trade-offs between seed number and seed size^22^,^23^ and models have been developed based on this relationship^37^. Bonal *et al*.^23^ describe a complex interaction between *Quercus ilex* and the weevil *Curculio elephas* in which both large seed crops and large acorns were effective in satiating weevils. Bonal *et al*.^23^ detected the commonly occurring negative relationship between the number and the size of the acorns, a relationship that precludes the possibility that the same tree could use both types of predator satiation. However, predatory weevils are not likely to distinguish individual trees but rather respond to the density of acorns produced by all of the trees in their environs. While individual trees may not be able to simultaneously satiate both large and small predators of acorns^23^, the population as a whole may be able to do so. In our study at the population level we specifically did not detect the seed size/number trade-off and this provides the possibility that *Q. schottkyana* may be able to satiate both small and large seed predators. Larger acorns were produced in both high and low production weeks and years. Larger acorns in low crop years may be more effective in satiating weevils because larger acorns increase the likelihood of embryo survival^5^ although effective satiation by larger acorns may be negated in years of lower acorn production.

These strategies have been classified as resistance and tolerance mechanisms^38^. Resistance mechanisms include anti-herbivore adaptations including acorn quality and these act to lessen acorn consumption. In contrast, tolerance mechanisms do not prevent acorn consumption but act to reduce acorn loss such as by the satiation of weevils by means of large but irregular seed crops^39^ and by annual variation in phenology. Most pre-dispersal predators are small, highly specialized insects, many of which develop inside the seed^40^. This practice of internal seed consumption gives seed size an uncertain role in influencing the likelihood of seed survival. Although larger seeds are more likely to suffer higher pre-dispersal predation than smaller seeds, larger seeds may have a higher probability of surviving predation because they can satiate the developing larvae before they reach the embryo, thus allowing continued development of the infested seeds.

We still do not know enough about our system to differentiate between at least four hypotheses: 1) the weevils differentially select the small acorns produced early in the season, 2) weevils attack the first acorns produced which happen to be small, 3) the early production of small acorns is a response by the oak to satiate weevils and permit production of larger acorns with higher germinability later in the season and 4) if late-season acorns are larger because they are produced late in the season, or if they are produced early season escape infestation and have longer time to develop. These hypotheses wait further testing.

### Interaction of abiotic and biotic factors

*Quercus schottkyana* appears to sacrifice acorns in the early season that are small and less likely to germinate even if they were not infested. This early dispersal of small infested acorns that are not viable could be a short-term defensive strategy against later weevil infestation^16^. Early abortion and drop of immature acorns is common for species in the Fagaceae and it usually occurs prior to the dispersal of the mature seeds. This is a general pattern by oak trees in response to seed predation by insects such as weevils^41^. An individual tree may save a considerable amount of energy if infested acorns are aborted early^37^; although 47% of acorns were attacked by weevils in the high crop year in 2006, because of the larger crop size, the absolute number of acorns that were not infested was still greater than that in the lower crop years such as 2011 and 2013. In lower crop years, weevils were not satiated by the early crop of small acorns and thus continue to infest acorns later into the season – this only occurred in 2011 and 2013 when few viable acorns were produced over the entire season. Acorns produced later in the year were both heavier and had higher germination rates. In normal to high acorn crop years, the trees may experience suitable abiotic conditions, acorn production is higher, weevils infest acorns in the early season, weevils are satiated and the remaining acorns germinate. These results highlight the importance of annual variation in production, size and quality of *Q. schottkyana* acorns in satiating and defending weevil infestation and consequently in successful acorn dispersal.

This leads us to ask why we see so few oak seedlings in this region. Every factor we measured points towards an oak-weevil system where, under most conditions, both are able to leave successful offspring. However, under unusually dry conditions (e.g. 2011), the relationship breaks down and the oak produces few viable acorns. In addition, those acorns that escape weevil infestation and are successfully dropped to the ground are then vulnerable to post-dispersal predation. In our study site, post-dispersal drought and infestation by various Coleoptera (Curculionidae) damage almost all the acorns on the ground by late May at the beginning of the wet season during which acorns can germinate^42^. In the Iberian Peninsula, up to 100% of the total acorn crop of *Q. ilex* was consumed by predators (wild boars and wood mice)^43^,^44^. Acorns therefore, move through a series of selective filters in which, if they are too small they may be aborted, while larger acorns may mature but become more susceptible to weevil infestation. Those that escape infestation and are dispersed i.e. dropped to the ground, are then subject to seed predators. We showed that larger acorns had a higher probability of germination yet there must be a trade-off because larger acorns are also more vulnerable to predators and dispersers^44^. Nevertheless, a few successful oak seedlings may be adequate to maintain these forests because, on average each individual adult tree simply has to replace itself. If on average an individual *Q. schottkyana* lives for 120 years, then on average, each individual has to leave only one successful offspring every 120 years. Successful reestablishment requires an episodic event or ecological crunch^45^, that greatly reduces the number of herbivores for one, or a few, years and during these herbivore-reduced conditions, seedlings may be able to grow past the vulnerable stages and successfully recruit to the community.

This region of southern China has experienced an increasing number of droughts and the recent drought in 2011 was especially severe. In addition to periodic stress from drought, since 1960 the number of rainy days has decreased significantly^46^. Thus, trees may be experiencing long-term stress from fewer rainy days, as well as more periodic stress events from yearly drought events. These more abnormal conditions can contribute to a breakdown of the reproduction process in an already stressed tree. Our data from the low acorn production years of 2011 and 2013, suggest that acorn production likely responds to different abiotic cues.

As weather becomes more variable, both acorns (as prey) and weevils (as predators) cannot be expected to alter their phenology simultaneously and are thus unlikely to remain synchronous. Changes in climate patterns may lead to the breakdown of population-level processes where oak and weevils respond differently to abiotic cues. Climatic variation may result in closely correlated responses and synchrony of population changes in some species^47^,^48^ and may influence population dynamics of one species through changes in food supplies and in another species through abiotic influences on reproduction. In the oak-weevil system we might expect that the highest rate of acorn infestation should occur at the peak of acorn production. However, our data show that peak infestation by weevils generally occurs just prior to peak acorn production and the maximum germination rate occurs well after peak infestation. This asynchronous timing and negative density dependent infestation has likely developed over many years allowing some large acorns to escape predation and survive to germinate while providing small acorns to weevils as food. There is yearly variability around these typical conditions but general phenological and negative density dependent processes allow predator and prey to coexist in relative stability. Changing climatic norms and increasing variability in weather intensity may break this pattern threatening both predator and prey.

The dynamics of populations interact in complex ways within a community responding to both abiotic and biotic events. In this study, external factors, primarily abiotic effects, can be extremely variable and exert a strong influence on the dynamics of acorn populations. The survival of acorns with the potential to recruit into the oak population are regulated by negative density dependent predation by weevils which themselves may be directly influenced by other external forces. Additional research needs to be done in our system to investigate if there is a direct link between large-scale climatic events, regional weather patterns and oak and weevil reproduction and their interactions. These links have been difficult to make^49^ and require many years of data and experimentation to address, but are essential if we are to understand how climate change will impact important ecosystems such as the one we study^48^. The negative density dependent relationship between acorn production and the rate of weevil attack is essential to understanding the reproductive life cycle of *Q. schottkyana*. However, weevils cannot be the only regulators of acorn seedling production. We are currently investigating the effects of other potential post-dispersal seed predators such as birds and small mammals, and other herbivores that may attack acorns after they germinate.

## Materials and Methods

### Study site

The study site was in a mixed forest north of Kunming, Yunnan Province, China (N25°01°, E102°41°). The forest has *Castanopsis orthacantha* Franch., *Lithocarpus dealbatus* (Hook. f. et Thoms. ex DC.) Rehd., *Quercus acutissima* Carruth., *Quercus aliena* Bl., *Alnus nepalensis* D. Don. Within the forest is a natural pure stand of *Q. schottkyana*, approximately 70 trees in an area of ~1 ha. from which we collected acorns. The region has a wet season (May-October) and a dry season (November-April) with an average 878 mm and 116 mm rainfall respectively^50^.

### The weevils

*Curculio* weevils usually lay their eggs in seeds from families such as Fagaceae, Betulaceae and Juglandaceae. Eighty-nine species of these seed beetles (Coleoptera: Curculionidae: Curculioninae: Curculionini) are known from China, 34 species from Yunnan Province and four species (*C. beverlyae, C. hobbsi, C. bidens, C. megadens*) in our study region^21^,^51^. Oak begins producing mature acorns usually in September and then they are attacked by adult weevils, which emerge in spring or late summer depending on the species. Female weevils climb to the tree canopy, mate, puncture the acorn and oviposit usually one egg^52^,^53^. However, subsequent oviposits by the same or other females can occur and multiple larvae can develop within the same acorn^53^. Once the larvae hatch inside the acorn they feed on the cotyledons until completing their development or until all the food resources of the acorn are depleted^53^. By this stage the acorns have been dropped to the ground. The larvae then bore an exit hole through the seed coat and over winter underground.

### The oak

*Quercus schottkyana* produces acorns annually and pollination occurs in the spring^33^. Acorns mature on the trees from late May to October and mature acorns fall to the ground between the end of August and mid-late November. Germination and seedling establishment mostly occurs through the following summer.

### Acorn production, infestation and germination

We counted the number of acorns produced between 2006 and 2014. Collections occurred daily, from the end of August to the end of November. To collect acorns we randomly placed fifty 30 cm diameter plastic containers throughout the site each year. Each basin contained 3 cm of sand to prevent acorns bouncing out. All of the acorns collected each week were packed in a loosely secured paper bag and stored at 5 °C until needed for further tests.

For the acorns collected each week, we determined the rates of acorn abortion and acorn infestation by weevils using cut tests^54^ on 30–90 acorns. Then we estimated the number of infested acorns in each week by multiplying the total number of acorns we collected in each week by the infestation rate in the week. Moisture content and dry mass of acorns were determined by drying 5–25 non-infested acorns at 103 °C for 17h^54^. Most acorns die when infested by weevils, so when we began germination tests in 2008 we used only acorns. From each weekly acorn collection two to eight replicates (dependent upon the number of acorns produced) of fifteen non-infested acorns were randomly chosen for germination tests. Acorns from each replicate were placed in a 90 mm Petri dish containing 1% agar in water and kept at 25 °C in a germination cabinet. We considered acorns germinated when there was a radical >2 mm long. Acorns were inspected every few days for 26 weeks to determine if germination had occurred. All aborted acorns had undeveloped or no visible embryos/cotyledons and were not able to germinate. When we were estimating the total number of germinated (viable) acorns in each week, we multiplied the total number of sound acorns (acorns which were not aborted and not infested by weevils) collected that week by the germination rates we obtained from germination tests of the sound acorns in that week.

### Data analysis

We used likelihood ratio tests on Generalized Linear Mixed Models (GLMMs) to account for differences in seed size, infestation and germination rates between either weeks or years. Models were built using week-level data with weekly and/or yearly covariates to account for pseudoreplication. For all tests, we used the most parsimonious random effect structure possible. When required, we compared the full model with different random effect structures using Akaike Information Criteria (AIC)^55^. We specified Poisson distributed errors for models of count data year, Gaussian errors for seed size data and binomially distributed errors for models describing infestation and germination rate data. We checked for over dispersion in all models using count data as a response. Cross validation procedures were used to test the sensitivity and specificity of our results. We randomly selected portions of our data set (90%) and iterated the analysis 100 times. All iterations provided the same test results as those obtained using the complete data set (see details in Supplement Tables S1, S2, S3). Because the locations for acorn collection were randomly placed each year, the yearly observations (estimated based on weekly production, infestation and germination data) are independent. We used likelihood ratio tests on Generalized Linear Models (GLMs) with binomial errors to determine if yearly weevil infestation rates and germination rates on acorns depended on acorn production. We determined the nature of density dependent relationships between infestation, germination and production by examining the sign of the slope of the relationships estimated in the respective models. When the relationship resulted in regulation of the population size of viable acorns, the density dependent relationship is negative, otherwise it is positive. All analyses were performed in R 3.3.1^56^ (function::lmer and function::glmer in package::lme4^57^ etc.).

## Additional Information

**How to cite this article**: Xia, K. *et al*. Pre-dispersal strategies by *Quercus schottkyana* to mitigate the effects of weevil infestation of acorns. *Sci. Rep.* **6**, 37520; doi: 10.1038/srep37520 (2016).

**Publisher’s note:** Springer Nature remains neutral with regard to jurisdictional claims in published maps and institutional affiliations.

## Supplementary Material

Supplementary Information
