# Supplementary material for: Pre-dispersal strategies by Quercus schottkyana to mitigate the effects of weevil infestation of acorns
Source: Sci Rep. 2016 Nov 22;6:37520. doi: 10.1038/srep37520 (PMC5118682; doi:10.1038/srep37520)

Supplementary Information

Pre-dispersal strategies by *Quercus schottkyana* to mitigate the effects of weevil infestation of acorns

Ke Xia, William L. Harrower, Roy Turkington, Hong-Yu Tan, Zhe-Kun Zhou

Supplemental Tables:

Table S1. Acorn production

1. Between-year variation in acorn production (Fig 1A):

Results of Likelihood ratio test to determine if the number of acorns produced was different between years. We used generalized linear mixed models with week number as a random effect and Poisson errors. We used week number as a random effect to account for the fact that acorn production is correlated between weeks. Acorn number was square root transformed and we tested for overdispersion in this model; there was none (SSQ residuals/residual df = 1.042, p = 0.223). Thus, we retained the Poisson distribution.

**Model**	**df**	**AIC**	**Deviance**	χ^2	**p**
Total number ~ 1	2	748.67	744.67		
Total number ~ Year	10	585.00	565.00	179.67	<0.001

Results of cross validation: the test was repeated 100 times using randomly select 90% of our data set, all the iterations achieved a p value of <0.001.

2. Acorn production over time

Results of Likelihood ratio test to determine if the number of acorns produced changes with week number. We used generalized linear mixed models with year as a random effect and Poisson errors. Because the relationship between acorns infection and week number is not monotonic, we used quadratic model. We used year as a random effect to account for

differences in production between years, and repeated measures over time. Acorn number was square root transformed and we tested for overdispersion in this model; there was none (SSQ residuals/residual df = 1.088, p = 0.143). Thus, we retained the Poisson distribution.

**Model**	**df**	**AIC**	**Deviance**	χ^2	**p**
Infested ~ 1	2	748.67	744.67		
Infested ~ Week number	4	570.14	562.14	182.52	<0.001

Results of cross validation: the test was repeated 100 times using randomly select 90%of our data set, all the iterations achieved a p value of <0.001.

3. The difference in acorn size between years:

Results of Likelihood ratio test to determine if acorns differ in size between years. We used generalized linear mixed models with week number as a random effect and Gaussian errors. We used week number as a random effect to account for the fact that acorn production is correlated between weeks.

**Model**	**df**	**AIC**	**Deviance**	χ^2	**p**
Size ~ 1	3	-13.059	-19.059		
Size ~ Year	11	-48.438	-70.438	51.38	<0.001

Results of cross validation: the test was repeated 100 times using randomly select 90% of our data set, all the iterations achieved a p value of <0.001.

4. *Acorn size over time (Fig 2A):*

Results of Likelihood ratio test to determine if acorn size differs with week number. We used generalized linear mixed models with week number as a fix effect and Gaussian errors. We used week number and year as a random effect to account for the fact that acorn production and size is correlated between weeks. We used year as a random effect to account for differences in production between years, and repeated measures over time.

**Model**	**df**	**AIC**	**Deviance**	χ^2	**p**
Size ~ 1	3	-15.525	-21.525		
Size ~ Week number	4	-60.045	-68.045	46.52	<0.001

Results of cross validation: the test was repeated 100 times using randomly select 90% of our data set, all the iterations achieved a p value of <0.001.

5. *Acorn size and production (Fig 2B):*

Results of Likelihood ratio test to determine if acorn size is dependent on the production of acorns. We used generalized linear mixed models with week number and year as a random effect and Gaussian errors. We used week number and year as random effects to account for the fact that acorn production and size is correlated between weeks. We used year as a random effect to account for differences in production between years, and repeated measures over time.

**Model**	**df**	**AIC**	**Deviance**	χ^2	**p**
Size ~ 1	4	-36.30	-44.30		
Size ~ Total number	5	-35.712	-45.712	1.41	0.2345

Results of cross validation: the test was repeated 100 times using randomly select 90%of our data set, all the iterations achieved a p value of >0.05.

Table S2. Infestation rates by weevils

1. Difference in infestation between years (Fig 1B):

Results of Likelihood ratio test to determine if the proportion of acorns infested is different between years. We used generalized linear mixed models with year as a fix effect and binomial errors. We used week number and year as a random effect to account for the fact that acorn production is correlated between weeks.

Model	df	AIC	Deviance	χ^2	p
Infested ~ 1	3	1273.9	1267.9		
Infested ~ Year	11	1246.8	1224.8	43.08	<0.001

Results of cross validation: the test was repeated 100 times using randomly select 90%of our data set, all the iterations achieved a p-value of <0.001.

2. Infestation over time (Fig 3A):

Results of Likelihood ratio test to determine if the proportion of acorns infested changes with week number. We used generalized linear mixed models with year as a random effect and binomial errors. Because the relationship between acorns infection and week number is not monotonic, we used quadratic model. We used year as a random effect to account for differences in production between years, and repeated measures over time.

Model	df	AIC	Deviance	χ^2	p
Infested ~ 1	2	2357.3	2353.3		
Infested ~ Week number	4	1735.2	1727.2	626.07	<0.001

Results of cross validation: the test was repeated 100 times using randomly select 90%of our data set, all the iterations achieved a p value of <0.001.

3. Infestation with production (Fig 3B):

Results of Likelihood ratio test to determine if the yearly proportion of acorns infested changes with yearly acorn density. We use generalized linear model with binomial errors. We assume the observations are independent between years, because the locations for acorn collection were randomly placed each year.

**Model**	**df**	**AIC**	**Deviance**	χ^2	**p**
Yearly Infested ~ 1	1	820.57	756.69		
Yearly Infested ~ Yearly acorn density	2	606.76	540.88	215.81	<0.001

Table S3. Acorn germination

1. Variation in germination between years (Fig 1C):

Results of Likelihood ratio test to determine if the proportion of acorns that germinated differed between years. We used generalized linear mixed models with week number as a random effect and binomial errors. We used week number as a random effect to account for the fact that acorn production is correlated between weeks.

**Model**	**df**	**AIC**	**Deviance**	χ^2	**p**
Germinated ~ 1	3	568.01	562.01		
Germinated ~ Year	8	546.96	530.96	31.06	<0.001

Results of cross validation: the test was repeated 100 times using randomly select 90%of our data set, all the iterations achieved a p value of <0.001.

2. Germination over time (Fig 3C):

Results of Likelihood ratio test to determine if the proportion of acorns that germinated changes with week number. We used generalized linear mixed models with year as a random effect and binomial errors. We used year as a random effect to account for differences in production between years, and repeated measures over time.

**Model**	**df**	**AIC**	**Deviance**	χ^2	**p**
Germinated ~ 1	2	900.61	896.61		
Germinated ~ Week Number	3	586.94	580.94	315.67	<0.001

Results of cross validation: the test was repeated 100 times using randomly select 90%of our data set, all the iterations achieved a p value of <0.001.

3. Germination and acorn density (Fig 3D):

Results of Likelihood ratio test to determine if the yearly proportion of acorns that germinated changes with yearly acorn density. We use generalized linear model with binomial errors. We assume the observations are independent between years, because the locations for acorn collection were randomly placed each year.

**Model**	**df**	**AIC**	**Deviance**	χ^2	**p**
Yearly Germinated ~ 1	1	369.49	327.97		
Yearly Germinated ~ Yearly acorn density	2	333.68	290.16	37.81	<0.001

Supplemental figures:

Figure S1. Data from Figure 2 A, B expanded and presented respectively as yearly effects; lines represent least squares fitted values and shaded areas are 95% confidence intervals. The points represent weekly acorn production for each year.

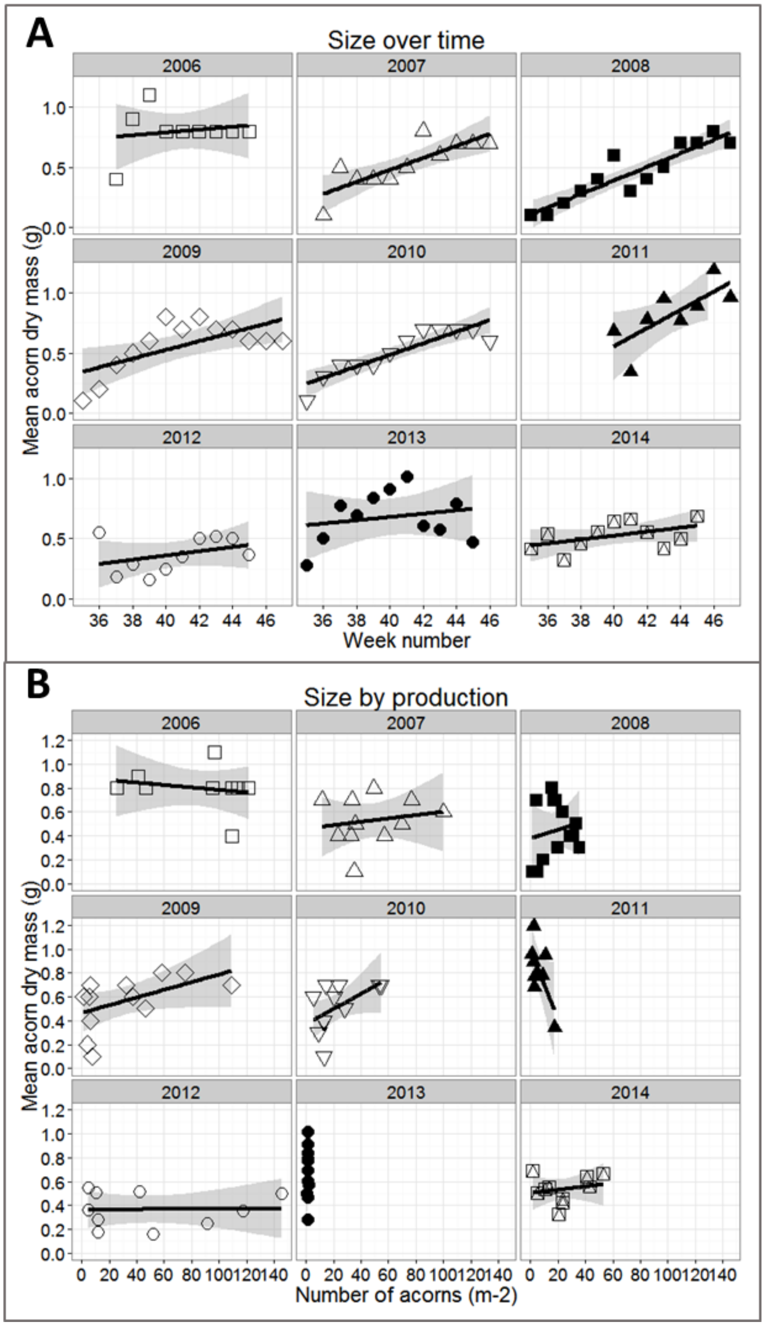

Figure S2. Data from Figures 3 A, B, C, D expanded and presented for each individual year; lines represent least squares fitted values and shaded areas are 95% confidence intervals. The points represent weekly acorn production for each year. Unlike averages across year where linear models were a better fit, quadratic models were fit to data on infestation over time.

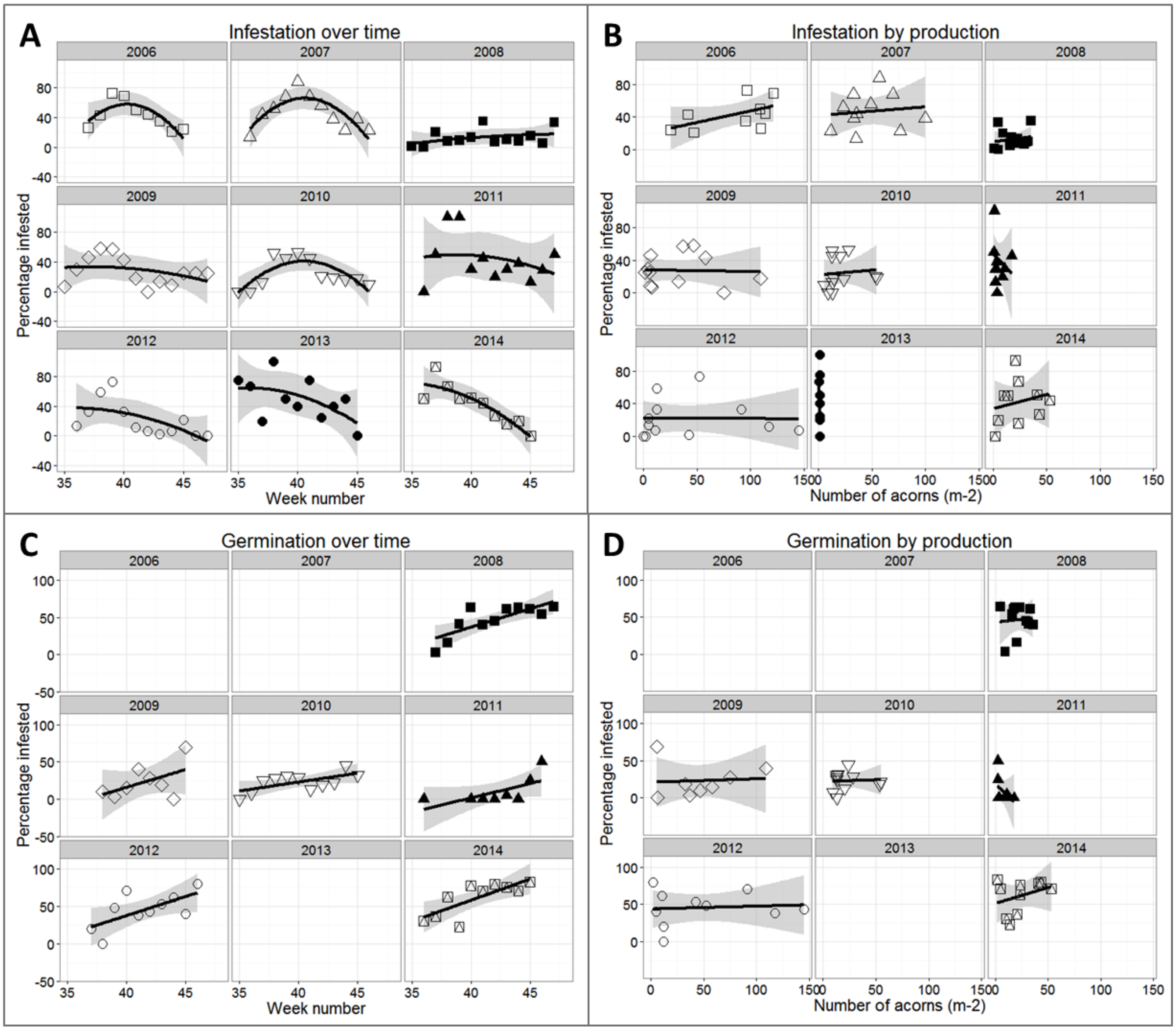

Supplement: Supplementary Information [file srep37520-s1.pdf]
